# Artemisinin-resistant *Plasmodium falciparum* clinical isolates can infect diverse mosquito vectors of Southeast Asia and Africa

**DOI:** 10.1038/ncomms9614

**Published:** 2015-10-20

**Authors:** Brandyce St. Laurent, Becky Miller, Timothy A. Burton, Chanaki Amaratunga, Sary Men, Siv Sovannaroth, Michael P. Fay, Olivo Miotto, Robert W. Gwadz, Jennifer M. Anderson, Rick M. Fairhurst

**Affiliations:** 1 Laboratory of Malaria and Vector Research, National Institute of Allergy and Infectious Diseases, National Institutes of Health, 12735 Twinbrook Parkway, Rockville, Maryland 20852, USA; 2 National Center for Parasitology, Entomology and Malaria Control, Phnom Penh 12101, Cambodia; 3 Biostatistics Research Branch, National Institute of Allergy and Infectious Diseases, National Institutes of Health, Rockville, Maryland 20852, USA; 4 Mahidol-Oxford Tropical Medicine Research Unit, Faculty of Tropical Medicine, Mahidol University, Bangkok 10400, Thailand; 5 Malaria Programme, Wellcome Trust Sanger Institute, Hinxton CB10 1SA, UK; 6 Medical Research Council (MRC) Centre for Genomics and Global Health, University of Oxford, Oxford OX3 7BN, UK

## Abstract

Artemisinin-resistant *Plasmodium falciparum* parasites are rapidly spreading in Southeast Asia, yet nothing is known about their transmission. This knowledge gap and the possibility that these parasites will spread to Africa endanger global efforts to eliminate malaria. Here we produce gametocytes from parasite clinical isolates that displayed artemisinin resistance in patients and *in vitro*, and use them to infect native and non-native mosquito vectors. We show that contemporary artemisinin-resistant isolates from Cambodia develop and produce sporozoites in two Southeast Asian vectors, *Anopheles dirus* and *Anopheles minimus*, and the major African vector, *Anopheles coluzzii* (formerly *Anopheles gambiae* M). The ability of artemisinin-resistant parasites to infect such highly diverse *Anopheles* species, combined with their higher gametocyte prevalence in patients, may explain the rapid expansion of these parasites in Cambodia and neighbouring countries, and further compromise efforts to prevent their global spread.

## Abstract (web summary)

It is unknown whether artemisinin-resistant malaria parasites from Southeast Asia can infect any African species of *Anopheles* mosquitoes and thus spread to Africa. Here, St. Laurent *et al.* show that artemisinin-resistant isolates from Cambodia can indeed infect the major African vector, *Anopheles coluzzii*.

## Graphical abstract

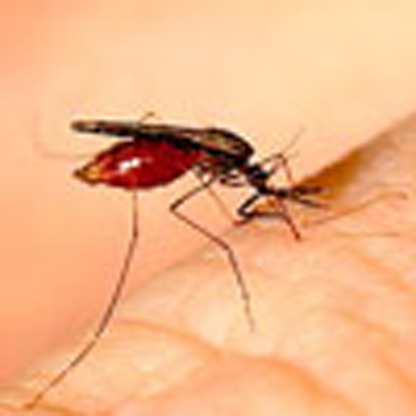

Artemisinin (ART) resistance in *P. falciparum* threatens to decrease the efficacy of all ART combination therapies (ACTs)^1^,^2^, severely compromising global efforts to eliminate this malaria parasite. Indeed, ART resistance has quickly led to the failure of dihydroartemisinin-piperaquine, the current frontline ACT for falciparum malaria in Western Cambodia^3^,^4^. Although ART-resistant *P. falciparum* parasites have emerged and rapidly expanded in Cambodia and other Greater Mekong Subregion (GMS) countries^5^,^6^,^7^,^8^,^9^,^10^, and may spread to Africa, nothing is known about their transmission to native or non-native mosquito vectors. Two recent findings in Western Cambodia, however, have important implications for the transmission of these parasites. First, patients with ART-resistant parasites more often present with gametocytemia, or develop gametocytemia while taking an ACT, than those with ART-sensitive parasites^9^, suggesting that ART-resistant parasites have increased transmission potential. Second, population genetics studies of *P. falciparum* clinical isolates have revealed that ART-resistant parasites often undergo population expansions, resulting in highly structured founder populations that are as differentiated from each other as they are from African parasites^11^,^12^. These founder populations display slow parasite clearance following ACTs in patients^11^,^12^, show resistance to ART in the Ring-stage Survival Assay *in vitro*^13^,^14^ and carry different *kelch13* (*K13*) mutations^8^,^12^ that confer ART resistance^15^,^16^.

The GMS hosts a great diversity of anopheline vectors capable of transmitting malaria^17^,^18^,^19^. The roles of these vectors in transmitting parasites from several ART-resistant founder populations and the ART-sensitive core population of Cambodia have not been investigated. Given that parasite clones within each ART-resistant founder population are nearly genetically identical^11^,^12^ and that *P. falciparum* strains can vary remarkably in their infectivity to *Anopheles* species *in vitro*^20^,^21^,^22^, we hypothesized that adaptation to native *Anopheles* species influences the spread of ART-resistant founder populations within the GMS and that failure to infect non-native vectors prevents their transmission in other regions. Characterizing the ability of ART-resistant parasites to infect vectors in Africa is particularly critical in assessing the risks of their spread to this continent, where most of the world’s malaria transmission, morbidity and mortality occur.

The anopheline fauna in Cambodia and other GMS countries includes a large number of vector species, entirely distinct from the relatively few vector species in malaria-hyperendemic settings of Africa. The diversity of *Anopheles* species in the GMS and the lack of knowledge about their bionomic characteristics complicate efforts to delimit, restrict and disrupt malaria transmission patterns in this region. Moreover, the predominantly outdoor-biting and outdoor-resting behaviors of these mosquitoes undermine vector control strategies that rely on indoor interventions, such as insecticide-treated bednet use and indoor residual spraying^17^, placing the burden of malaria control primarily on clinical interventions that have repeatedly failed owing to drug resistance^19^. Although *An. dirus* is believed to be the primary malaria vector in Cambodia, and other species including *An. minimus*, *An. maculatus* and *An. barbirostris* are considered secondary vectors, the current roles of these vectors in transmitting malaria have not been adequately defined.

To investigate the current transmission dynamics of *P. falciparum*, we produced gametocytes from parasite clinical isolates that displayed ART resistance or sensitivity in patients^6^,^11^,^12^ and *in vitro*^13^,^14^, and used them to infect native and non-native mosquito vectors. Here we show that contemporary ART-resistant isolates from Cambodia develop and produce sporozoites in two Southeast Asian vectors, *An. dirus* and *An. minimus*, and the major African vector, *An. coluzzii* (formerly *An. gambiae* M). The ability of ART-resistant parasites to infect such diverse anophelines, combined with their higher gametocyte prevalence in patients^9^, may be contributing to the rapid expansion of these parasites in Cambodia and other GMS countries. These findings pose serious challenges to eliminating ART-resistant malaria from Southeast Asia and preventing its spread to Africa.

## Results

Despite the alarming potential of ART resistance spreading throughout the GMS and to Africa, we still do not know whether Cambodian founder populations can infect major Southeast Asian malaria vectors or *An. coluzzii* and *An. gambiae*^23^, the two major African vectors. To address these knowledge gaps, we used a standard membrane feeding assay to assess whether nine Cambodian *P. falciparum* clinical isolates can infect five different *Anopheles* species. *P. falciparum* mosquito-infective gametocytes were induced and matured in culture for 2 weeks and fed to mosquitoes using glass feeders with an artificial membrane. Fed mosquitoes were dissected 1 week later to evaluate oocyst infection of their midguts (a standard measurement of mosquito infection), and the salivary glands of a subset of these mosquitoes were examined an additional 1 week later for the presence of human-infective sporozoites. Parasite isolates were tested in recently adapted colonies of *An. dirus* from Pursat Province, Western Cambodia, and *An. coluzzii* from Theirola, Mali; a long-term-adapted colony of *An. minimus* from Western Thailand; and two long-term-adapted *Anopheles stephensi* Nijmegen and *An. gambiae* G3 lines. These latter two lines are commonly used in laboratory investigations because they are highly permissive to infection by multiple *P. falciparum* strains, a phenotype for which they were artificially selected decades ago. These five vector species are highly evolutionarily distinct, having diverged from each other more than 30 million years ago^24^.

**Figure 1:**
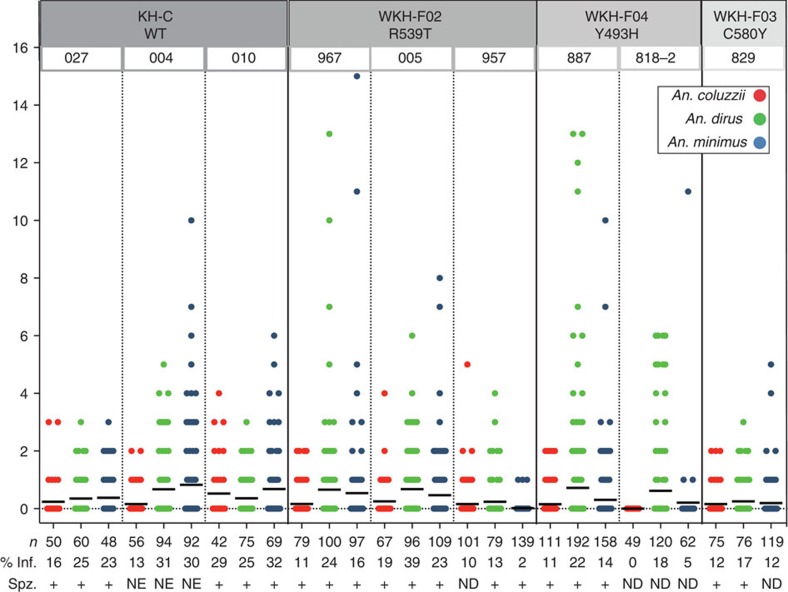
Infection of different *Anopheles* species by Cambodian *P. falciparum* clinical isolates. Three ART-sensitive parasites carrying WT *K13* alleles (KH-C/WT) and six ART-resistant parasites from three Western Cambodian founder populations carrying different mutant *K13* alleles (WKH-F02/R539T, WKH-F04/Y493H and WKH-F03/C580Y) were used to infect malaria vectors of Africa (*An. coluzzii*) and Southeast Asia (*An. dirus* and *An. minimus*) in parallel. Infection intensity was measured by counting the number of parasite oocysts per mosquito midgut in individual mosquitoes 8 days after they were fed gametocyte-infected erythrocytes. Each dot represents the oocyst count in a single mosquito midgut; black bars indicate the mean number of oocysts per midgut for all mosquitoes dissected. At least four independent feeds were performed for each parasite–mosquito combination. *n*, number of fed mosquitoes checked for oocysts; % Inf., proportion of fed mosquitoes with ≥1 oocyst; Spz., sporozoites were found in the salivary glands of a subset of mosquitoes dissected on day 14 after feeding (+), or their presence could not be determined (ND), typically owing to low infection rates, or were not examined (NE).

For mosquito infection experiments, we selected three parasites from the ART-sensitive core population (KH-C) in Ratanakiri Province, Eastern Cambodia, which carry the wild-type (WT) *K13* allele, and six parasites from three ART-resistant founder populations in Pursat, which carry different mutant *K13* alleles: WKH-F02/R539T (*n*=3), WKH-F04/Y493H (*n*=2) and WKH-F03/C580Y (*n*=1)^11^,^12^. Most of these isolates were previously found to be either ART sensitive or ART resistant in patients^6^, *in vitro*^14^ or both (Table 1). With a single exception, all nine parasite isolates infected all five mosquitoes tested (Fig. 1 and Supplementary Fig. 1). Consistent with previous studies of laboratory-based infections of Southeast Asian and African vectors with *P. falciparum* clinical isolates^22^,^25^,^26^,^27^, mosquitoes infected with our Cambodian *P. falciparum* isolates showed relatively low oocyst counts: one or two oocysts per infected midgut were typically seen, whereas more than five oocysts per infected midgut were uncommon and infection prevalence was usually <30% (Fig. 1 and Supplementary Figs 1 and 2). We found no indication that any of the five mosquito lines were refractory to infection; that is, we did not observe melanized parasites in the midguts of non-infected mosquitoes or infected mosquitoes with low numbers of oocysts.

**Figure 2:**
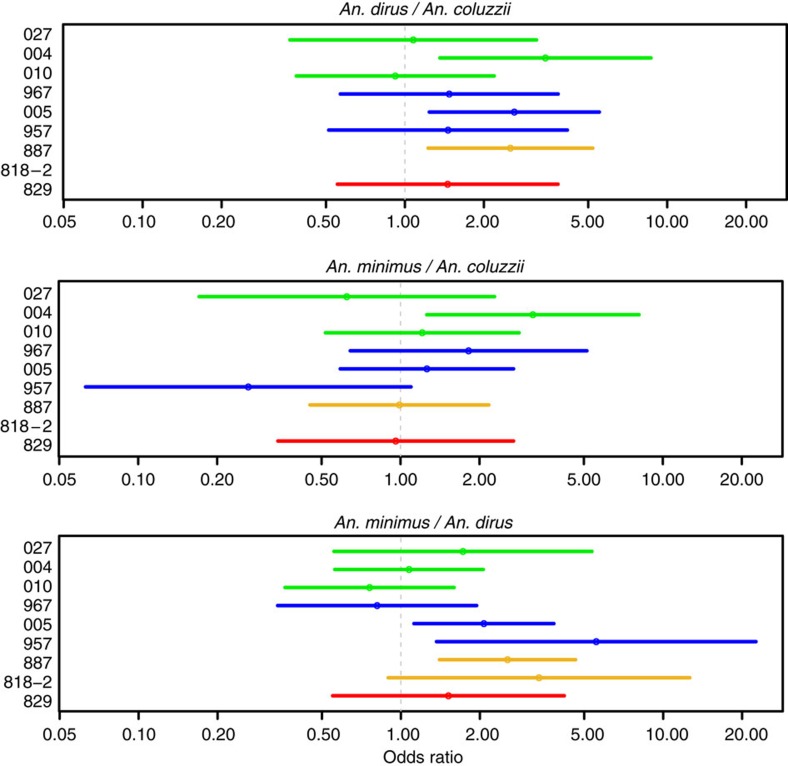
Relative infectivity of *P. falciparum* clinical isolates to *An. dirus*, *An. minimus* and *An. coluzzii*. Estimates (points) and 95% confidence intervals (lines) for odds ratios of any oocyst infection, done by a separate logistic regression for each isolate that controls for replicate feeds. The top panel shows the odds for infection of *An. dirus* over the odds for *An. coluzzii*, the middle panel shows the odds for *An. minimus* over the odds for *An. coluzzii* and the bottom panel shows the odds for *An. minimus* over the odds for *An. dirus.* Isolates are colored according to population: KH-C/WT (green), WKH-F02/R539T (blue), WKH-F04/Y493H (yellow) and WKH-F03/C580Y (red).

Supplementary Fig. 2 shows differential oocyst prevalence between five mosquito species infected with each of nine parasite isolates. For some isolates (004, *P*=0.0120; 005, *P*=0.0137; 957, *P*=0.0300; 887, *P*=0.0021; analysis of deviance), there were significant differences in infection prevalence between *An. dirus*, *An. minimus* and *An. coluzzii* (Fig. 2). Only the effect for isolate 887, however, remained significant after adjusting for multiple comparisons (*P*_adj_=0.019). For a different set of isolates (010, *P*=0.0327; 967, *P*=0.0022, *P*_adj_=0.0157; 887, *P*=0.0144; 818-2, *P*=0.0011, *P*_adj_=0.0087; 829, *P*<0.0001, *P*_adj_<0.0001), there were significant differences in infection prevalence between *An. stephensi* Nijmegen and *An. gambiae* G3 (Supplementary Fig. 3). These differential mosquito species effects on infection prevalence do not appear to cluster by founder population and we see no significant clustering (*P*=0.85 for Fig. 2 and *P*=0.26 for Supplementary Fig. 3; permutation test). These differences in infection prevalence suggest that highly related parasites within a given founder population possess genetic differences that mediate differential infection of anophelines. When considering only infected mosquitoes, we found that no isolate showed a significant difference in oocyst intensity between *An. dirus*, *An. minimus* and *An. coluzzii* (overall *P*=0.1651; analysis of variance test of interaction) or between *An. stephensi* Nijmegen and *An. gambiae* G3 (overall *P*=0.2411), but this lack of significance may be attributable to low infection rates.

Subsets of mosquitoes were also examined 2 weeks after parasite infection for the presence of salivary-gland sporozoites, the human-infective stage of *P. falciparum* in natural settings. We observed salivary-gland sporozoites in 153 of 216 (71%) mosquitoes, including *An. dirus*, *An. minimus* and *An. coluzzii*, infected with ART-resistant isolates from all three founder populations (Fig. 1). In 90% of preparations, we found sporozoites to be motile—an indication of viability. Other subsets of infected mosquitoes showed no sporozoites, but this is probably explained by our observations of unruptured oocysts and inability to detect sporozoites due to low infection rates (that is, there were no infected mosquitoes in the subset checked for sporozoites). All seven isolates we examined produced sporozoites in *An. stephensi* Nijmegen and *An. gambiae* G3 (Supplementary Fig. 1). Infections of *An. dirus* and *An. coluzzii* with two ART-resistant isolates (887 and 967) showed comparable numbers of salivary-gland sporozoites per infected female mosquito (Supplementary Fig. 4).

## Discussion

Collectively, our data indicate that all nine ART-resistant and ART-sensitive parasites readily infect and produce sporozoites in highly diverse *Anopheles* species. Although infections with multiple independently reared colonies of each *Anopheles* species may be necessary to determine their species-specific susceptibility to these parasite isolates, our preliminary study has two important strengths. First, all of the ART-resistant clinical isolates we studied were clonal members of founder populations, which are the most relevant sources of the parasites that initially emerged and subsequently spread in the natural setting of Western Cambodia—the epicenter of ART resistance in Southeast Asia. Second, we tested two recently colonized vectors—*An. dirus* from Western Cambodia and *An. coluzzii* from Mali—that are arguably more relevant to the current potential spread of ART-resistant parasites in Southeast Asia and Africa, respectively, than the laboratory-selected, highly permissive *An. stephensi* Nijmegen and *An. gambiae* G3 lines that are commonly used in infection studies.

**Figure 3:**
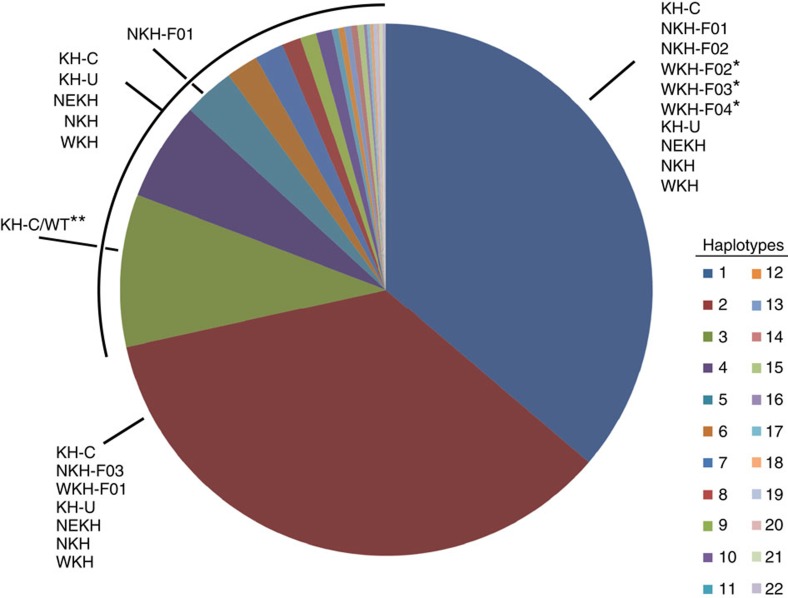
Distribution of *Pfs47* haplotypes among Cambodian *P. falciparum* clinical isolates. The *Pfs47* alleles of 516 parasite isolates were genotyped against the 3D7 reference genome (ver. 3). This procedure identified 22 different haplotypes, which are listed in order of decreasing prevalence in our sample collection (Supplementary Tables 1 and 2). The *Pfs47* haplotypes of parasites from the core population of Eastern Cambodia (KH-C) and multiple founder populations of Western (WKH), Northern (NKH) and Northeastern (NEKH) Cambodia are indicated. The six ART-resistant isolates we used in our study belong to the WKH-F02, WKH-F04 and WKH-F03 founder populations (*), all of which possess the same *Pfs47* haplotype 1 (Supplementary Tables 1 and 2). The three ART-sensitive isolates we used belong to the KH-C/WT population (**) and have the same *Pfs47* haplotype 3 (Supplementary Tables 1 and 2). It is noteworthy that *Pfs47* haplotype 1 is present in four of five ART-resistant founder populations, whereas all 22 *Pfs47* haplotypes are present in ART-sensitive isolates (Supplementary Table 2).

Our data show that several contemporary parasite isolates from Cambodia differentially infect *Anopheles* species in a laboratory setting. Although elucidating the molecular basis of this finding requires further investigation, we have excluded a possible role for polymorphism in the *Pfs47* gene (*PF3D7_1346800*), which was recently shown to mediate the differential infectivity of the NF54, GB4 and 7G8 *P. falciparum* strains in the *An. stephensi* Nijmegen and *An. gambiae* G3 lines^28^,^29^. All six ART-resistant isolates we tested possess the same *Pfs47* haplotype (Fig. 3 and Supplementary Table 1), the most common among 22 haplotypes identified in our recent collection of 516 Cambodian parasite isolates (Supplementary Table 2). Similarly, the three ART-sensitive isolates we tested share the same *Pfs47* haplotype (Fig. 3 and Supplementary Table 1), the third most common in our parasite collection (Supplementary Table 2).

As all nine parasites infected nearly every anopheline we tested, it is possible that these two *Pfs47* haplotypes do not strongly restrict parasite–mosquito compatibility in Cambodia and may not prevent the spread of similar isolates within Africa. As ART-resistant founder populations circulate in areas of high anopheline diversity, they may have been naturally selected on a genetic background of *Pfs47* alleles (or other genetic determinants) that collectively confer the ability to infect most native anophelines and thus to efficiently spread. As all ART-resistant founder populations possess either of the two most common *Pfs47* haplotypes we have observed in Cambodia (Fig. 3 and Supplementary Tables 1 and 2), the lack of *Pfs47* polymorphism among our ART-resistant isolates may simply reflect founder effects. This is because *Pfs47* is in close proximity to *K13* on chromosome 13 and thus is likely to be genetically linked with the *K13* haplotype in founder populations.

In summary, our finding that all five mosquito lines are susceptible to parasite infection suggests that native and non-native vectors are likely to be competent in transmitting ART-resistant and ART-sensitive *P. falciparum* populations beyond Cambodia. Detailed studies of field-relevant parasites and vectors, including whole-genome sequence analyses of multiple Southeast Asian *P. falciparum* isolates^11^,^12^,^30^ and recently colonized *Anopheles* species^24^, will undoubtedly help unravel the complex genetic and behavioral determinants of malaria transmission in regions where vector species are poorly characterized. Our finding that highly differentiated ART-resistant parasites infect highly diverse *Anopheles* species unveils a significant unanticipated challenge to the elimination of malaria in Southeast Asia and the prevention of ART-resistant malaria in Africa.

## Methods

### Parasite culture

The *P. falciparum* isolates we used in this study are as follows: three parasites from the ART-sensitive core population (KH-C) in Ratanakiri that carry the WT *K13* allele and six parasites from three ART-resistant founder populations in Pursat that carry different mutant *K13* alleles: WKH-F02/R539T (*n*=3), WKH-F04/Y493H (*n*=2) and WKH-F03/C580Y (*n*=1)^11^,^12^ (Table 1). *P. falciparum* clinical isolates were adapted and maintained in O^+^ human erythrocytes (Interstate Blood Bank Inc., Memphis, TN) in RPMI 1640 medium containing HEPES, 25 mM sodium bicarbonate, 50 mg l^−1^ hypoxanthine and 10% heat-inactivated human serum at 1–2% parasitemia. Gametocytogenesis was induced by initiating cultures at high parasitemia and then diluting them to 3–6% parasitemia at 5% hematocrit in 20 or 30 ml of culture medium. Cultures were incubated at 37 °C in an atmosphere of 5% O_2_, 5% CO_2_ and 90% N_2_, and medium was changed daily for 14–16 days.

### Mosquitoes

The *An. dirus* A colony was isolated from Veal Veng District, Pursat Province, Western Cambodia, in July 2011. Unlike most *An. dirus* A colonies, this colony mates freely in our insectary. The identity of this colony was confirmed by sequencing the ribosomal DNA ITS2 region. Its rDNA sequence was 100% identical to a previously reported *An. dirus* A rDNA sequence^31^. The *An. minimus* A colony was obtained from the Malaria Research and Reference Reagent Resource Center as part of the BEI Resources Repository, NIAID, NIH: *An. minimus* MINIMUS1, MRA-729, deposited by M.Q. Benedict. This colony was isolated in Mae Sot, Western Thailand, in 1990. The *An. coluzzii* (formerly *An. gambiae* M) colony was isolated from Theirola, Mali, in November 2012. The identity of this colony was confirmed by genotyping via PCR and *Hha*-I digestion^32^. The *An. stephensi* Nijmegen colony was obtained from Joep Meuwissen, Catholic University of Nijmegen, The Netherlands, in 1985. The *An. gambiae* G3 colony, a laboratory line comprising a mixture of *An. gambiae* M and S forms, was obtained from George Davidson, London School of Hygiene and Tropical Medicine, in 1973.

### Mosquito feeds

Mosquitoes were reared at low density (200 per 26 × 32 cm tray or 500 per 38 × 52 cm tray) at 27 °C and 75% humidity on a 12:12 light–dark cycle. Three- to 6-day-old mosquitoes, prepared at a density of 30–60 per pint container, were artificially fed using glass feeders heated to 37 °C and covered with paraffin film or hog gut membranes. Feeders were loaded with 400 μl of feed mixture diluted to ∼0.15% mature stage-V gametocytemia. Feed mixtures were fed to all five *Anopheles* colonies in parallel and each feed was done at least four times. The presence of exflagellating gametocytes in the feed mixture was confirmed before and after feeding.

### Mosquito dissections

Parous female mosquitoes were dissected on day 8 post infection and their midguts stained with 0.2% mercurochrome in water and examined under a compound microscope. A subset of mosquitoes infected with each isolate was set aside and examined on day 14 post infection for the presence of sporozoites in salivary glands, which were dissected and ruptured under a cover slip and examined by microscopy at × 40 magnification. The number of sporozoites in the salivary glands of infected female mosquitoes was estimated using a hemocytometer. Salivary glands were dissected from females with at least one ruptured oocyst at 16–18 days post infection, placed in 40 μl of PBS and pipetted repeatedly to release sporozoites into solution. Ten microlitres of this sporozoite suspension were loaded onto a hemocytometer, sporozoites in each 4 × 4 grid were counted and standard calculations were performed to estimate the number of sporozoites per infected female mosquito.

### Statistical analysis

Prevalence effects were modelled using logistic regression that controlled for replicate feed effects. Replicates that added no information about differential mosquito species infection rates (for example, replicates where no oocysts were observed in any mosquito species) were removed from that analysis. Inference used Wald’s confidence intervals for individual odds ratio effects and analysis of deviance for *P*-values of overall effect. The notation *P*_adj_ refers to Holm’s adjusted *P*-values used to adjust for the fact that nine isolates were tested^33^. To test for the clustering founder effect, we used a permutation test, where the test statistic is the sum of the squared errors of the log odds ratio effects within the founder group in Fig. 2 and Supplementary Fig. 3, and we permuted the founder membership of the isolates. We calculated *P*-values by Monte Carlo with 10,000 replications. Tests on the oocyst intensity used linear regression on the log counts, including main effects for flask (and hence isolate) and mosquito vector, and interaction effects between isolate and vector, with the overall test of interaction done by analysis of variance. Statistical analyses were done using R (Version 3.2.0). *P*-values <0.05 were deemed significant.

### Genotyping and population genetics

Deep-sequencing data for 603 *P. falciparum* clinical isolates contributed by the authors were obtained by the MalariaGEN *Plasmodium falciparum* Community Project (http://www.malariagen.net/projects/parasite/pf). Full details of sample preparation, sequencing and genotyping are given elsewhere^12^. Briefly, DNA extracted from leukocyte-depleted venous blood was sequenced using the Illumina HiSeq platform and the resulting paired short reads were assembled and quality filtered to produce genotype calls for 681,587 high-quality variations. Genotype calls were supported by at least five sequencing reads, with at least two reads required to call individual alleles. These genotype calls were used to call *K13* genotypes using a custom procedure^12^ and to define *Pfs47* haplotypes, which could be reconstructed without missingness in 516 samples. Parasites were classified into core and founder populations by a multi-stage clustering technique based on ancestry analysis, as previously described^12^. All nucleotide read sequences were deposited in the European Nucleotide Archive database (http://www.ebi.ac.uk/ena), where they are publicly accessible; a list of European Nucleotide Archive accession codes is given in Supplementary Data 1.

## Additional information

**How to cite this article:** St. Laurent, B. *et al.* Artemisinin-resistant *Plasmodium falciparum* clinical isolates can infect diverse mosquito vectors of Southeast Asia and Africa. *Nat. Commun.* 6:8614 doi: 10.1038/ncomms9614 (2015).

## Supplementary Material

Supplementary Information Supplementary Figures 1-4 and Supplementary Tables 1-2

Supplementary Data 1 European Nucleotide Archive accession codes for 603 samples used in the present analysis. The samples and data used in this publication form part of the MalariaGEN *Plasmodium falciparum* Community Project (http://www.malariagen.net/projects/parasite/pf) and the Pf3k Project (http://www.malariagen.net/projects/parasite/pf3k). Specifically, these data belong to partner studies led by Dr. Rick Fairhurst. The contributing investigators and data producers have agreed to release the sequence data of these samples in the expectation that other researchers will find them useful and will respect the data-sharing principles that are guided by the principles of the Fort Lauderdale Agreement, (www.genome.gov/Pages/Research/WellcomeReport0303.pdf), and are available on the MalariaGEN website (http://www.malariagen.net/projects/parasite/pf3k/terms-of-use).

## Figures and Tables

**Table 1:** Characteristics of Cambodian *P. falciparum* clinical isolates used to infect *Anopheles* species.

**Isolate**	**Population**	***K13*** **allele**	**Origin**	**Year**	**Parasite clearance half-life (h)**	**RSA**^**0–3h**^ **survival rate (%)**
KH001-027	KH-C	WT	Ratanakiri	2011	4.6	ND
KH003-004	KH-C	WT	Ratanakiri	2011	1.6	ND
KH003-010	KH-C	WT	Ratanakiri	2011	2.5	ND
967	WKH-F02	R539T	Pursat	2010	6.0	48.9
KH001-005	WKH-F02	R539T	Pursat	2011	6.2	ND
957	WKH-F02	R539T	Pursat	2010	6.9	28.1
887	WKH-F04	Y493H	Pursat	2009	8.3	6.8
818-2	WKH-F04	Y493H	Pursat	2010	8.0	3.5
829	WKH-F03	C580Y	Pursat	2009	8.2	ND

ART, artemisinin; *K13*, *kelch13*; ND, not determined; RSA, Ring-stage Survival Assay; WT, wild type.

Three ART-sensitive parasites from a core population (KH-C) in Eastern Cambodia and six parasites from three ART-resistant founder populations (WKH-F02, WKH-F04 and WKH-F03) in Western Cambodia are listed. For each parasite, the *K13* allele, province of origin, year of collection, parasite clearance half-life in patients and % survival value in the RSA^0–3h^ *in vitro* are shown.
