# Corrigendum: Artemisinin-resistant *Plasmodium falciparum* clinical isolates can infect diverse mosquito vectors of Southeast Asia and Africa

**DOI:** 10.1038/ncomms10345

**Published:** 2016-01-08

**Authors:** Brandyce St. Laurent, Becky Miller, Timothy A. Burton, Chanaki Amaratunga, Sary Men, Siv Sovannaroth, Michael P. Fay, Olivo Miotto, Robert W. Gwadz, Jennifer M. Anderson, Rick M. Fairhurst

*Nature Communications* 6: Article number: 861410.1038/ncomms9614 (2015); Published: 10202015; Updated: 01082016.

In the first paragraph of the Results section in this Article, the *An. gambiae* G3 line is incorrectly stated to have been artificially selected for permissiveness to infection by multiple *P. falciparum* strains. The following sentence is correct: ‘These latter two lines are highly permissive to infection by multiple *P. falciparum* strains, a phenotype for which *An. stephensi* Nijmegen was artificially selected' (Feldmann and Ponnudurai). In addition, in the second paragraph of the Discussion section, a reference to a recent report that the *Pfs47* gene mediates the differential infectivity of three *P. falciparum* strains incorrectly states that this was demonstrated in the *An. stephensi* Nijmegen and *An. gambiae* G3 lines. This should have read: ‘Although elucidating the molecular basis of this finding requires further investigation, we have excluded a possible role for polymorphism in the *Pfs47* gene (*PF3D7_1346800*), which was recently shown to mediate the differential infectivity of the NF54, GB4 and 7G8 *P. falciparum* strains in the *An. gambiae* G3-L35 line'.

Feldmann, A. M. & Ponnudurai, T. Selection of *Anopheles stephensi* for refractoriness and susceptibility to *Plasmodium falciparum*. *Med. Vet. Entomol.* **3,** 41-52 (1989).

