# Supplementary material for: Artemisinin-resistant Plasmodium falciparum clinical isolates can infect diverse mosquito vectors of Southeast Asia and Africa
Source: Nat Commun. 2015 Oct 20;6:8614. doi: 10.1038/ncomms9614 (PMC4616032; doi:10.1038/ncomms9614)
Supplement: Supplementary Information — Supplementary Figures 1-4 and Supplementary Tables 1-2 [file ncomms9614-s1.pdf]

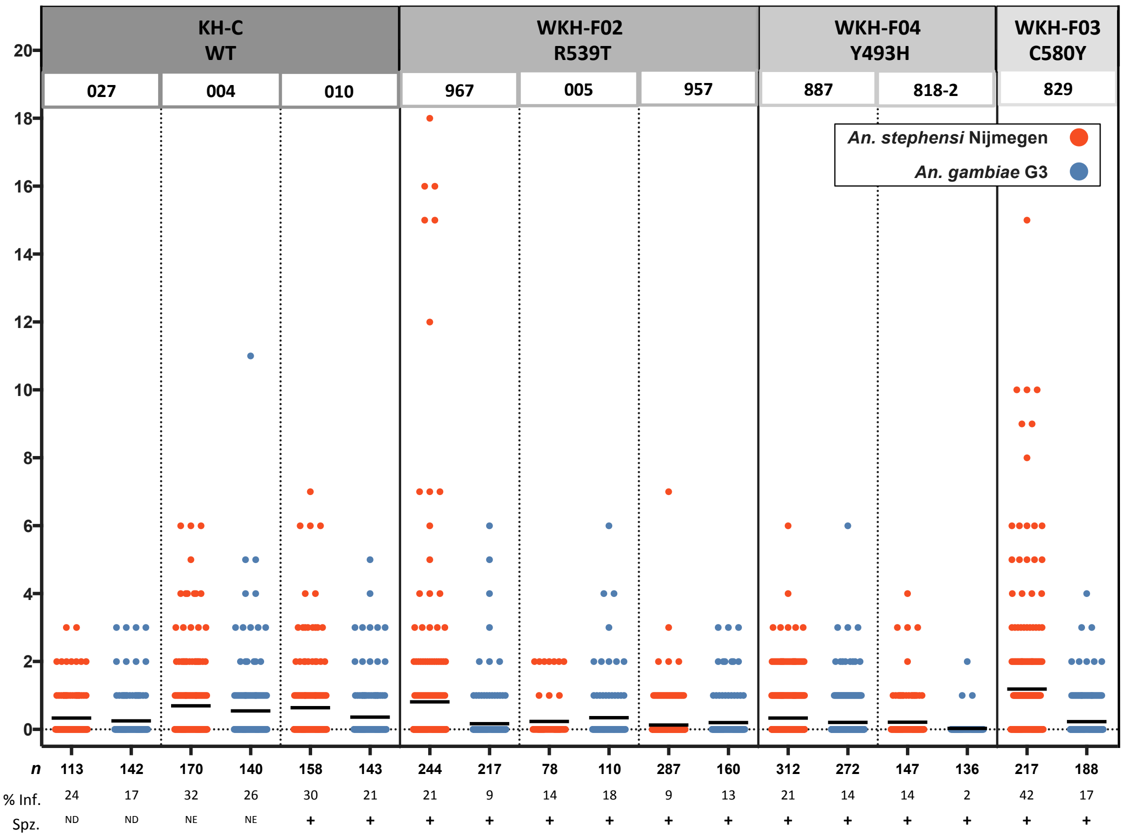

Supplementary Fig. 1. Infection of two highly permissive *Anopheles* lines by Cambodian *Plasmodium falciparum* clinical isolates. Three ART-sensitive parasites carrying wild-type *K13* alleles (KH-C/WT), and six ART-resistant parasites from three Western Cambodian founder populations carrying different mutant *K13* alleles (WKH-F02/R539T, WKH-F04/Y493H, and WKH/F03/C580Y), were used to infect two *Anopheles* lines (*An. stephensi* Nijmegen and *An. gambiae* G3) that were artificially selected to be highly permissive to infection with multiple *P. falciparum* strains. In these experiments, parasite infections of *An. stephensi* Nijmegen and *An. gambiae* G3 were performed in parallel with those of *An. dirus*, *An. minimus*, and *An. coluzzii* (**Fig. 1**). Infection intensity was measured by counting the number of parasite oocysts per mosquito midgut in individual mosquitoes eight days after they were fed gametocyte-infected erythrocytes. Each dot represents the oocyst count in a single mosquito midgut; black bars indicate the mean number of oocysts per midgut count for all mosquitoes dissected. At least four independent feeds were performed for each parasite-mosquito combination. *n*, number of fed mosquitoes checked for oocysts; % Inf., proportion of fed mosquitoes with ≥ 1 oocyst; and Spz., sporozoites were found in the salivary glands of a subset of mosquitoes dissected on day 14 after feeding (+), or their presence could not be determined (ND), typically due to low infection rates, or were not examined (NE).

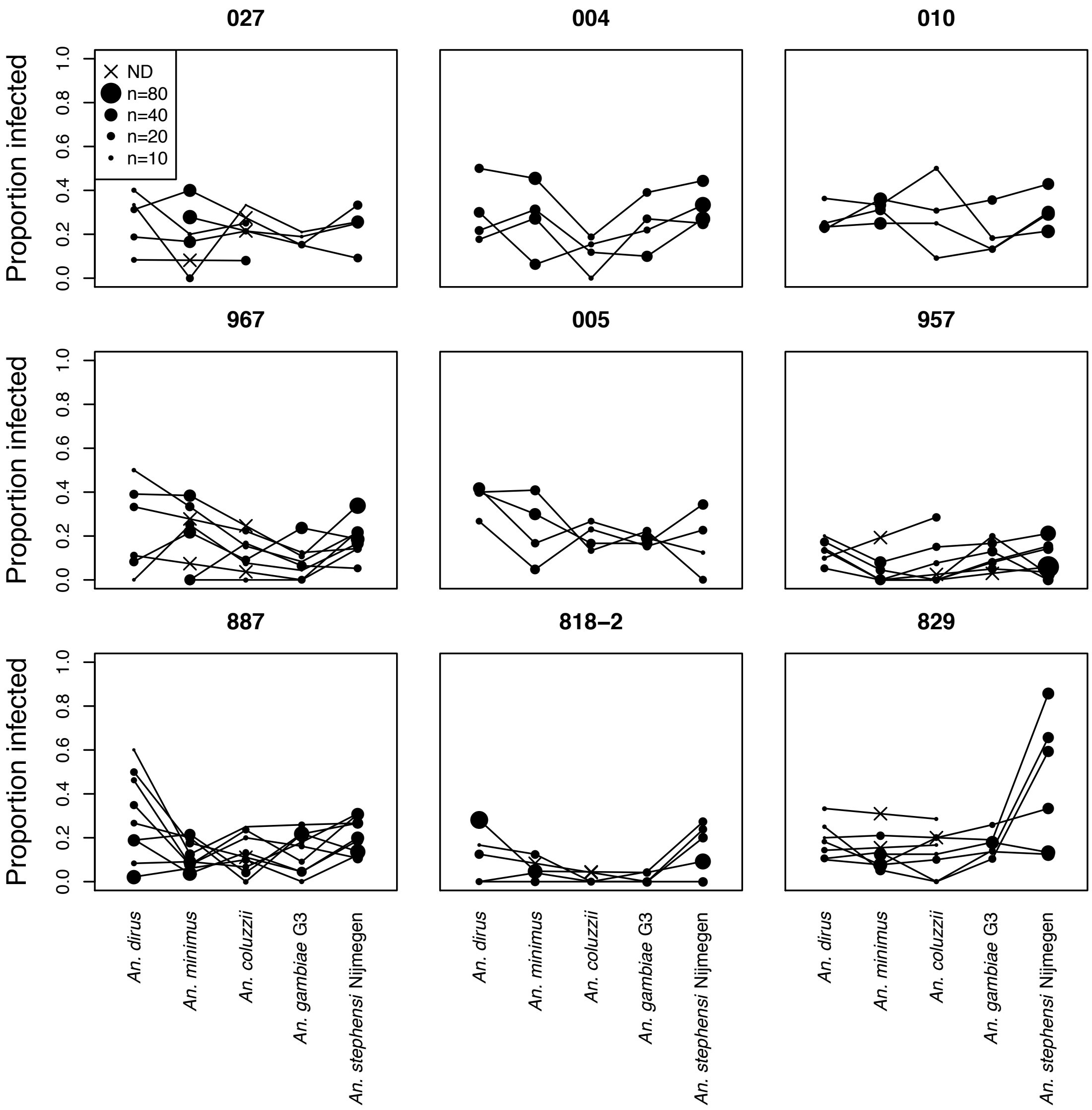

Supplementary Fig. 2. Proportion of five different *Anopheles* species infected by each of nine Cambodian *Plasmodium falciparum* clinical isolates. A line connects the proportions of fed mosquitoes infected by an individual parasite isolate in a single replicate feed. In full replicates, all five *Anopheles* species were fed at the same time. In partial replicates, an "X" denotes no mosquitoes dissected. The size of the points are proportional to the number of fed mosquitoes dissected.

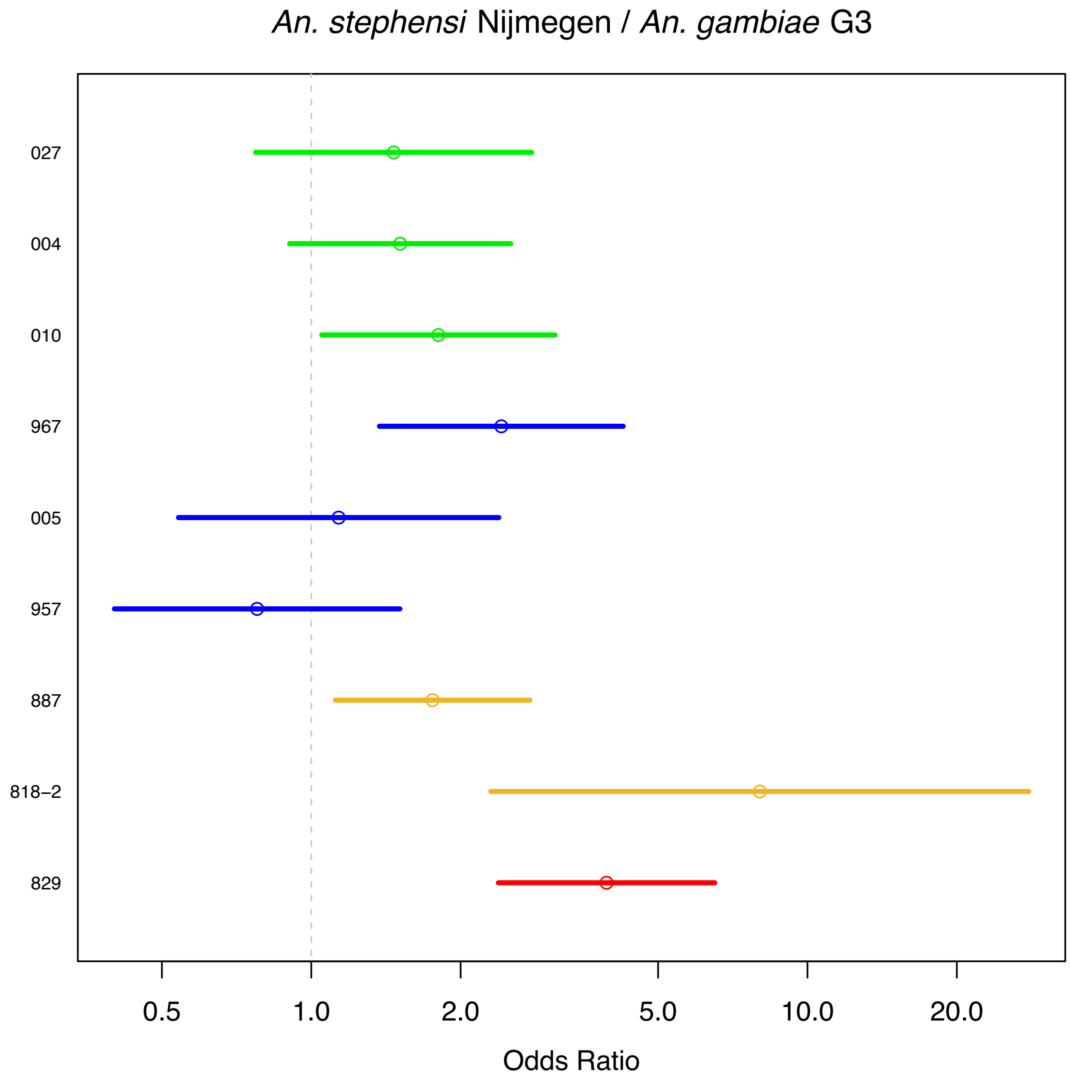

Supplementary Fig. 3. Relative infectivity of parasite isolates between *An. stephensi* Nijmegen and *An. gambiae* G3. Estimates (points) and 95% confidence intervals (lines) for odds ratios of any oocyst infection, done by a separate logistic regression for each isolate that controls for replicate feeds. The figure shows the odds for infection of *An. stephensi* Nijmegen over *An. gambiae* G3. Isolates are colored according to population: KH-C (green), WKH-F02 (blue), WKH-F04 (yellow), and WKH-F03 (red).

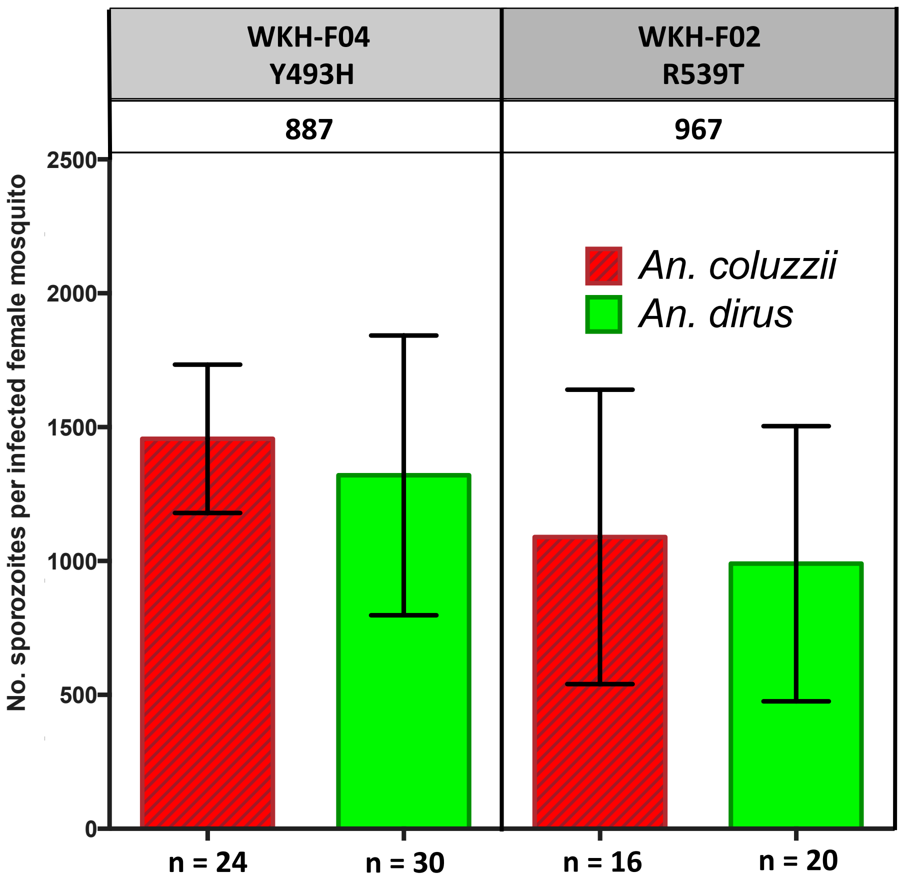

Supplementary Fig. 4. Sporozoite counts of ART-resistant *P. falciparum* isolates in *An. coluzzii* and *An. dirus.* Salivary glands dissected from infected female mosquitoes were used to estimate the number of sporozoites per infected female mosquito for two ART-resistant isolates, 887 and 967, in recently colonized *An. coluzzii* from Mali (red) and *An. dirus* from Cambodia (green). Bars represent the s.e.m. The mean number of sporozoites per individual infected mosquito is not significantly different between *An. coluzzii* and *An.* dirus or between parasite isolates in the same mosquito species (unpaired t-test $p > 0.5$).

Haplotype	1878898 F433V	1878899 432I	1878919 I426L	1878956 413I	1878980 K405N	1879381 N272Y	1879453 I248L	1879477 L240I	1879488 T236I	1879524 I224N	1879614 P194H	1879638 I186K	1880032 E55K	1880113 L28I
**1**	A	A	T	G	T	A	A	T	A	T	T	A	C	T
**2**	A	A	T	G	T	A	A	T	A	T	T	A	T	T
**3**	A	A	T	G	T	T	A	T	A	T	T	T	T	T
**4**	A	G	T	G	T	T	A	T	A	T	T	T	T	T
**5**	A	G	T	G	T	A	A	G	A	A	T	A	C	T
**6**	A	G	T	G	T	T	A	T	A	T	T	T	C	T
**7**	A	A	A	G	T	A	A	T	A	T	T	A	C	T
**8**	C	A	A	G	T	A	A	T	A	T	T	A	C	T
**9**	A	A	T	G	T	T	A	T	A	T	T	T	C	T
**10**	A	G	T	A	T	T	A	T	A	T	T	T	T	T
**11**	A	A	T	G	T	T	A	G	A	A	T	T	T	T
**12**	A	A	T	G	T	A	A	T	A	T	T	T	T	T
**13**	A	A	T	G	T	T	A	T	A	T	T	A	T	T
**14**	A	A	T	G	G	A	A	T	A	T	T	A	C	T
**15**	A	G	T	G	T	A	A	T	A	T	T	A	C	T
**16**	A	A	T	G	T	T	A	T	A	T	T	A	C	T
**17**	A	A	A	G	T	A	A	T	A	T	T	A	T	T
**18**	A	G	T	G	T	A	A	T	A	T	T	T	T	T
**19**	C	A	A	G	T	A	A	T	A	T	T	T	C	T
**20**	A	G	T	G	T	A	A	T	A	T	T	A	T	T
**21**	C	A	T	G	T	A	A	T	A	T	T	A	C	T
**22**	A	A	T	G	T	A	A	T	A	A	T	A	C	T

Supplementary Table 1. *Pfs47* haplotypes in 516 Cambodian *Plasmodium falciparum* clinical isolates. Parasite isolates were genotyped against the 3D7 reference genome (ver. 3). This procedure identified 22 different *Pfs47* haplotypes, which are listed in order of decreasing prevalence (**Supplementary Fig. 4, Supplementary Table 2**). Nucleotides that differ from the reference sequence are shown against a colored background. Column headings indicate the genomic position on chromosome 13, and the amino acid change caused by the alternative allele on the 3D7 genome, in the absence of other mutations.

Haplotype	KH-C	NEKH	NKH-F01	NKH-F02	NKH-F03	NKH	WKH-F01	WKH-F02	WKH-F03	WKH-F04	WKH	KH-U	Total
1	35	12	3	8		8		**12**	**3**	**12**	25	69	187
2	18	7			10	8	43				35	61	182
3	**21**	7				1					2	17	48
4	20	1										10	31
5	1		6			3						6	16
6	8		1			1							10
7	2											7	9
8											2	4	6
9	1										3	1	5
10						2						3	5
11						1						1	2
12						2							2
13	2												2
14	2												2
15	1											1	2
16	1												1
17	1												1
18	1												1
19	1												1
20		1											1
21											1		1
22												1	1
Total	115	28	10	8	10	26	43	12	3	12	68	181	516

Supplementary Table 2. Distribution of *Pfs47* haplotypes in 516 Cambodian *Plasmodium falciparum* clinical isolates. The number of parasites carrying each *Pfs47* haplotype listed in Supplementary Table 1 is shown here. KH-C, core population; NKH-F01 to NKH-F03, founder populations in Northern Cambodia; WKH-F01 to WKH-F04, founder populations in Western Cambodia; KH-U, unclassified (did not meet criteria for membership of either core or founder populations); NEKH, Northeastern Cambodia; NKH, Northern Cambodia; WKH, Western Cambodia (NEKH, NKH, and WKH samples were not included in the founder population analysis). The artemisinin-resistant founder populations are shown against a colored background. The parasites we used in the infection experiments belong to the four populations shown in red.
